# Effect of Polysaccharide Extracted From *Gynostemma Pentaphyllum* on the Body Weight and Gut Microbiota of Mice

**DOI:** 10.3389/fnut.2022.916425

**Published:** 2022-05-26

**Authors:** Shiwei Li, Yingna Wang, Weipeng Dun, Wanqing Han, Tao Ning, Qi Sun, Zichao Wang

**Affiliations:** ^1^College of Life Sciences and Agronomy, Zhoukou Normal University, Zhoukou, China; ^2^College of Food Science and Technology, Henan University of Technology, Zhengzhou, China; ^3^School of Biological Engineering, Henan University of Technology, Zhengzhou, China; ^4^College of Life Sciences, Chongqing Normal University, Chongqing, China

**Keywords:** *Gynostemma pentaphyllum*, polysaccharide, mice, body weight, gut microbiota

## Abstract

Researchers have investigated the role of polysaccharides in disease treatment *via* gut microbiota regulation but ignore their function in disease prevention and physique enhancement. In this work, a *Gynostemma pentaphyllum* polysaccharide (GPP) was tested by methyl thiazolyl tetrazolium (MTT) assay and proved to be safe to Caco-2 cells. Animal experiments showed that the administration of GPP for 3 weeks decreased the body weight gain of mice from 15.4 ± 1.7 to 12.2 ± 1.8 g in a concentration-dependent manner. Analysis of short-chain fatty acids (SCFAs) indicated that GPP increased the levels of acetate, propionate, butyrate, and total SCFAs in the cecum contents of normal mice. Furthermore, GPP improved the species richness and abundance in the gut microbiota but reduced the Firmicutes/Bacteroidetes ratio from 0.8021 to 0.3873. This work provides a basis for incorporating GPP into diet to prevent or mitigate the occurrence of obesity *via* gut microbiota regulation.

## Introduction

With lifestyle changes and living standard improvement, unreasonable diet has become an important factor that directly influences human health. Three high-metabolic syndromes, namely, hyperglycemia, hyperlipidemia, and hypertension caused by high sugar and high fat diet, have become a serious threat to physical health, quality of life, and national economy development. Therefore, the irreplaceable diet in people's daily life could be used as an entry point by modifying functional properties to improve health and prevent the occurrence and development of diseases. A daily intake of dietary fiber could reduce the harmful effects of stroke, coronary disease, type 2 diabetes, and colon cancer (1). Active substances in diet have good prevention and treatment effects against nutritional diseases and may include functional polysaccharides from natural medicinal and edible plants (2). Functional polysaccharides derived from edible plants can be used as food supplements that can improve people's diet by scavenging oxygen free radicals and inhibiting lipid peroxidation, α-glucosidase activity, α-amylase activity, promoting insulin secretion, and regulating the metabolism of glucose and lipids (3).

Gut microbiota, a community of microorganisms in the gut, plays an important role as a bridge among food nutrition and health. The gut microbiota not only participates in nutrition, metabolism, and immune regulation but also in the occurrence and development of nutritional, metabolic, and immune diseases. Cani (4) found that depending on the source of amino acids, gut microbiota can mitigate metabolic diseases *via* improving or altering metabolism of body and the corresponding metabolites. Zhao et al. (5) suggested that dietary fibers could promote gut microbiota to produce diversity and abundance of short-chain fatty acids (SCFAs) and diminish production of metabolically detrimental compounds such as indole and hydrogen sulfide, thus alleviating type 2 diabetes *via* improving hemoglobin A1c levels and increasing glucagon-like peptide-1 production. Mardinoglu et al. (6) reported that low-carbohydrate diet alleviated non-alcoholic fatty liver might *via* increasing the folate-producing *Streptococcus* in gut microbiota and serum folate concentrations, thus down-regulating of the fatty acid synthesis pathway and up-regulating of folate-mediated one-carbon metabolism and fatty acid oxidation pathways. Furthermore, certain effects have been achieved by gut microbiota transplantation, in which the feces of a healthy donor are transplanted into patients to reconstruct a new gut microbiota and regulate health (7). Gut microbiota has gradually developed into a biomarker of human health, disease prevention, and immune regulation.

Polysaccharides can affect nutrition and health by regulating the gut microbiota. For instance, Sun et al. (8) demonstrated that polysaccharides extracted from plants could alleviate circadian rhythm disorders and related psychiatric disorders by regulating the gut microbiota. Liu et al. (9) verified that mannan-oligosaccharide could alleviate the cognitive and behavioral disorders of mice with 5xFAD Alzheimer's disease by regulating the gut microbiota–brain axis. Mo et al. (10) found that insoluble yeast β-glucan could alleviate the high-fat diet-induced obesity of mice by regulating the gut microbiota and its metabolites. Li et al. (11) suggested that alginate oligosaccharides can protect against fumonisin B1-induced intestinal damage by promoting gut microbiota homeostasis. In our previous work, we found that a *Gynostemma pentaphyllum* polysaccharide (GPP) could adjust the high blood sugar level of diabetic mice to the normal level (12). When the absorption mechanisms of a *Ganoderma lucidum* polysaccharide (GLP) were investigated *in vivo* and *in vitro*, we found that GLP entered the body and reached the posterior part of the intestinal tract to participate in gut microbiota metabolism (13). However, the role of GPP is improving body health through gut microbiota regulation remains unclear. Therefore, the toxicity of GPP was first investigated; after which, the effect of GPP on body weight and short-chain fatty acids (SCFAs) in the intestine and gut microbiota of mice was analyzed.

## Materials and Methods

### Materials

*Gynostemma pentaphyllum* (Thumb) Makino herb was bought from a local drugstore of Zhengzhou (China). Caco-2 cells (HTB037) were acquired from the American Type Culture Collection and stored in our laboratory (13). Specific pathogen-free Kunming male mice (SCXK 2017-0002, 18–22 g) were bought from the Experimental Animal Center of Henan province (China). Dimethyl sulfoximine (DMSO), Dulbecco's modified Eagle's medium (DMEM), and 3-(4,5-Dimethylthiazol-2-yl)-2,5-bromo diphenyltetrazolium were purchased from Sigma-Aldrich (Shanghai, China). Fetal bovine serum, penicillin, and streptomycin were bought from Beyotime Biotechnology Co., Ltd., (Shanghai, China). Ethyl alcohol, sodium chloride, ethyl ether, sulfuricacid, calcium chloride, acetate, propionate, butyrate, and other chemical reagents were bought from Sinopharm Chemical Reagent Co., Ltd., (Beijing, China).

### Preparation of GPP

Extraction, collection, and purification of the *Gynostemma pentaphyllum* polysaccharide (GPP) were conducted based on previous methods (12), no protein or nucleic acid were detected in the obtained GPP. Then, the freeze-dried GPP powder was used in following experiments.

### Toxicity Analysis of GPP

Toxicity of GPP was determined using methyl thiazolyl tetrazolium (MTT) method against Caco-2 cells by using a previously reported method with some modifications (14). The freeze-dried GPP powder was dissolved and stirred in DMEM to concentrations of 50, 100, 200, 400, and 800 μg/ml, respectively. Meanwhile, DMEM solution without dissolving GPP was used as blank control. Caco-2 cells were cultured in DMEM with fetal bovine serum (10%, v/v), penicillin (100 U/ml), and streptomycin (100 μg/ml) in a humidified 5% CO_2_ incubator (Series 8000 WJ, Thermo Fisher Scientific, Waltham, MA, United States) at 37°C. During cultivation, Caco-2 cells were digested and counted intermittently. Upon reaching 2 × 10^4^ cells/ml, Caco-2 cells were transferred into 96-well plates and incubated in the CO_2_ incubator for 24 h at 37°C. Each well was added with 100 μl of GPP solution with different concentrations by using a pipette (Eppendorf, Germany) and cultured for another 24 h. The solution was then added with 20 μl of 3-(4,5-dimethylthiazol-2-yl)-2,5-bromo diphenyltetrazolium (5 mg/ml) and incubated for 4 h. The cell supernatant was discarded, and the insoluble crystals in Caco-2 cells were dissolved by adding 150 μl of DMSO. Absorbance was recorded using a microplate reader (BIO-RAD, Hercules, CA, United States) at 490 nm.

### Experimental Design and Sample Collection

Thirty mice (20 ± 1.0 g) were acquired from the Experimental Animal Center of Henan province (China) and fed in a specific-free pathogen environment, with temperature of 22–26°C, humidity of 60 ± 5%, and light: dark cycle of 12: 12 h. All animal procedures were performed in accordance with the guidelines for care and use of laboratory animals of Henan University of Technology. Experiments were approved by the Animal Ethics Committee of Henan University of Technology. Mice were fed according to routine feeding procedure and given food and drink freely for the first 7 days to acclimatize. After which, the mice ate (components of mice food are as follows: water 95 g/kg, crude protein 195.5 g/kg, crude fat 47.3 g/kg, coarse fiber 21.4 g/kg, crude ash 54.6 g/kg, calcium 13.6 g/kg, and total phosphorus 8.09 g/kg) and drank (tap water is boiled and cooled for use) freely throughout the whole experiment except for administration process. Then, twenty-four robust mice were selected and randomly divided into four groups, with six mice each (*n* = 6): control group (NC), administered with 0.4 ml of distilled water orally once a day; based on the preliminary experiment, experimental groups, orally administered with 100 μg/ml (2 g/kg) GPP (low-dose group, LOW), 400 μg/ml (8 g/kg) GPP (middle-dose group, MID), and 800 μg/ml (16 g/kg) GPP (high-dose group, HIG). If a single administration of 0.4 ml was difficult, then two consecutive doses of 0.2 ml were given. The night before administration, mice were fasted but drank freely. Mouse experiments lasted for 21 days, and the mice weight was measured weekly. The experimental mice were then fasted for 12 h and sacrificed by cervical dislocation. The contents in cecum were collected and stored at −80°C for further analysis.

### Detection of Short-Chain Fatty Acids

In brief, 0.2 g of cecum contents were dissolved in 1 ml of deionized water, vibrated for 3 min, and processed with ultrasonic wave for 30 min. The solution was added with 0.2 ml of 50% H_2_SO_4_ and 2 ml of diethyl ether and mixed acutely for 30 min. The mixture was placed in a water bath shaker at room temperature at 250 *r*/min for 30 min and centrifuged at 8,000 × *g* and 4°C for 10 min. The organic supernatant was collected and mixed with 1.0 g of anhydrous calciumchloride for 10 min to remove water. The supernatant was filtered against a 0.22 μm organic-based filter membrane, and SCFAs in organic layer were analyzed according to previously reported methods (14).

### Gut Microbiota Analysis

Gut microbiota was extracted and detected according to method reported previously with some modifications (14). Briefly, the total microbial DNA in the bacteria of collected cecum contents was extracted by using DNA extraction kit. V3-V4 hypervariable region of 16S rRNA was used as primer, and the primer sequences were 338F (5′-ACTCC TACGG GAGGC AGCAG-3′) and 806R (5′-GGACT ACHVG GGTWT CTAAT-3′). The amplification process was repeated three times for each sample, and the PCR products were recovered by using 2% (w/v) agarose gel electrophoresis. Meanwhile, AxyPrep DNA Gel Extraction Kit was used to purify the PCR products. After which, the PCR products were sent to Majorbio Co., Ltd., China (Shanghai, China) for gut microbiota analysis using Illumina MiSeq platforms according to the operation manual.

### Statistical Analysis

All data were expressed as mean of three parallel experiments. Analysis of variance (ANOVA) was performed using Origin software (Origin Pro 8.5).

## Results and Discussion

### Toxicity Analysis of GPP

Previously, a polysaccharide of GPP was successfully extracted from *Gynostemma pentaphyllum* herb, the monosaccharide composition of which was rhamnose, arabinose, galactose, glucose, xylose, mannose, galacturonic acid and glucuronic acid with molar ratio of 4.11: 7.34: 13.31: 20.99: 1.07: 0.91: 4.75: 0.36. Meanwhile, weight-average molecular weight (Mw) and number-average molecular weight (Mn) of GPP were 4.070 × 10^4^ and 3.924× 10^4^ Da, and its polydispersity (Mw/Mn) was 1.037 (12). However, due to geography and climate difference, monosaccharide composition and molecular weight of GPP were different from polysaccharides extracted from different *G. pentaphyllum* herbs reported by other researchers (15, 16).

**Figure 1:**
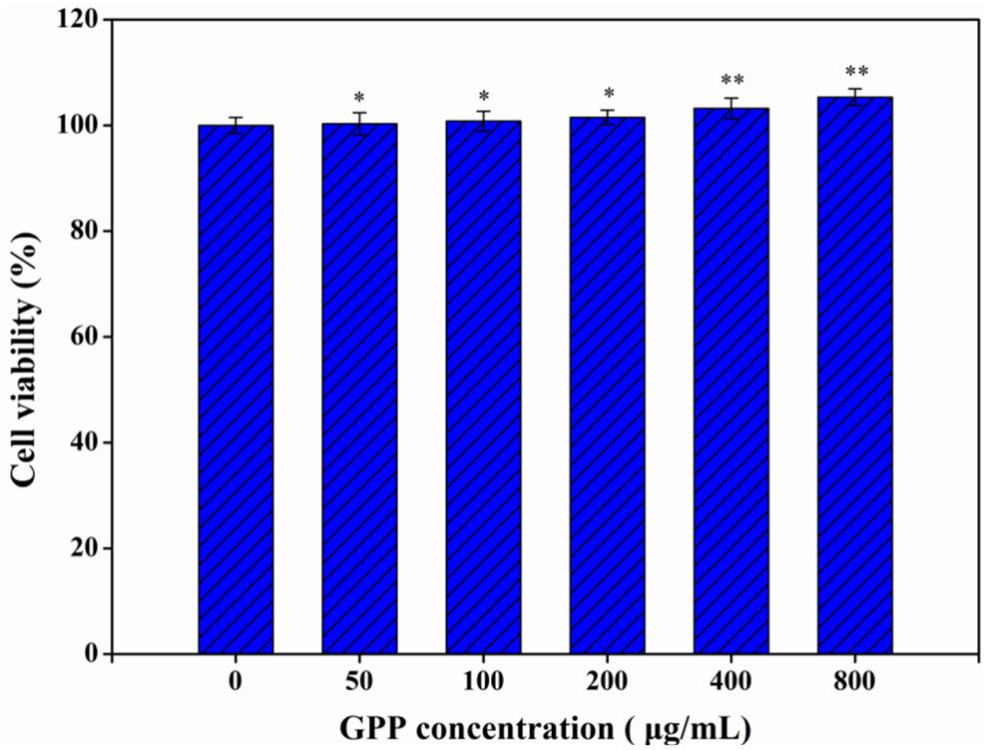
Toxicity of GPP to Caco-2 cells by MTT assay. Data are expressed as mean ± SD (*n* = 3). Significance was determined through ANOVA, *P < 0.05, **P < 0.01.

Meanwhile, GPP could reduce the blood sugar level of diabetic mice to normal *via* α-glucosidase inhibition and anti-inflammatory effects (3, 12), but the safety of GPP has been neglected. The toxicity of GPP to Caco-2 cells was detected by MTT assay. Figure 1 shows that none of the detected GPP concentration affected the viability of Caco-2 cells, indicating that GPP was safe to Caco-2 cells. Similarly, Li et al. (17) reported that *G. pentaphyllum* polysaccharide had no specific cytotoxicity to Hs-68 cells. Li et al. (18) also found that *Eucommia ulmoides* polysaccharide was non-toxic to Raw 264.7 macrophages. In our previous works, we found that *Ganoderma lucidum* polysaccharide and *Chaetomium globosum* CGMCC 6882 exo-polysaccharide were not toxic to Caco-2 cells (13, 14). In the present work, experimental mice showed no symptoms of death or disease, suggesting the safety of GPP to mice, which was in consistent with the safe result of GPP to Caco-2 cells. Furthermore, He et al. (19) reported that polysaccharide produced by *Streptomyces Virginia* H03 was non-toxic and did not kill mice even at 500 mg/kg/day.

### Influence of GPP on Mouse Body Weight

With improvement of living standards, changes in lifestyle, and reduction in physical activity, an increasing number of people suffer from obesity. Except for proper exercise and diet control, reasonable diet structure is an effective means to prevent and alleviate obesity (20). As shown in Table 1, the weights of mice in the control and experimental groups increased gradually with extension of feeding time. However, the body weight gain of experimental mice was negatively correlated with GPP concentration, that is, it decreased from 15.4 ± 1.7 g (CON) to 14.3 ± 1.9 g (LOW), 13.7 ± 1.6 g (MID), and 12.2 ± 1.8 g (HIG). Hence, GPP could be added in food or diet to prevent and mitigate obesity. Similarly, Chen et al. (21) reported that *Pueraria lobata* polysaccharide had similar body weight regulation effects. In contrast to the present study, Zhao et al. (22) found that *Auricularia auricular* polysaccharide did not affect the body weight. Furthermore, Yin et al. (23) found that non-polysaccharide substance of resveratrol could regulate the loss of body weight. GPP regulates body weight possibly by three mechanisms: inhibiting enzyme activity and nutrient absorption rate (24); adjusting the composition and proportion of gut microbiota, especially Firmicutes/Bacteroidetes (F/B) ratio (25); and degradation and conversion of GPP into SCFAs (26). The exact mechanism of GPP in regulating body weight is still being investigated.

**Table 1:** Influence of GPP on body weight of mice.

**Mice weight**	**CON**	**LOW**	**MID**	**HIG**
0 day (g)	19.3 ± 1.3^a^	19.8 ± 1.2^a^	20.1 ± 1.8^a^	19.5 ± 2.2^a^
7 day (g)	24.0 ± 2.1^b^	24.5 ± 1.9^b^	25.2 ± 1.6^b^	24.4 ± 2.1^b^
14 day (g)	28.2 ± 1.9^c^	28.1 ± 1.8^c^	28.5 ± 1.5^c^	27.6 ± 1.7^c^
21 day (g)	31.7 ± 2.0^d^	31.3 ± 1.6^d^	31.2 ± 1.8^d^	30.1 ± 1.5^d^
28 day (g)	34.7 ± 1.8^e^	34.1 ± 2.1^e^	33.8 ± 2.0^e^	31.7 ± 1.9^d^
Weight gain (g)	15.4 ± 1.7^a^	14.3 ± 1.9^b^	13.7 ± 1.6^b^	12.2 ± 1.8^c^

*CON, control group; LOW, 100 μg/ml GPP; MID, 400 μg/ml GPP; HIG, 800 μg/ml GPP. Data are expressed as mean ± SD (n = 6). Different letters represent significant differences, P < 0.05*.

### Effect of GPP on SCFAs

**Figure 2:**
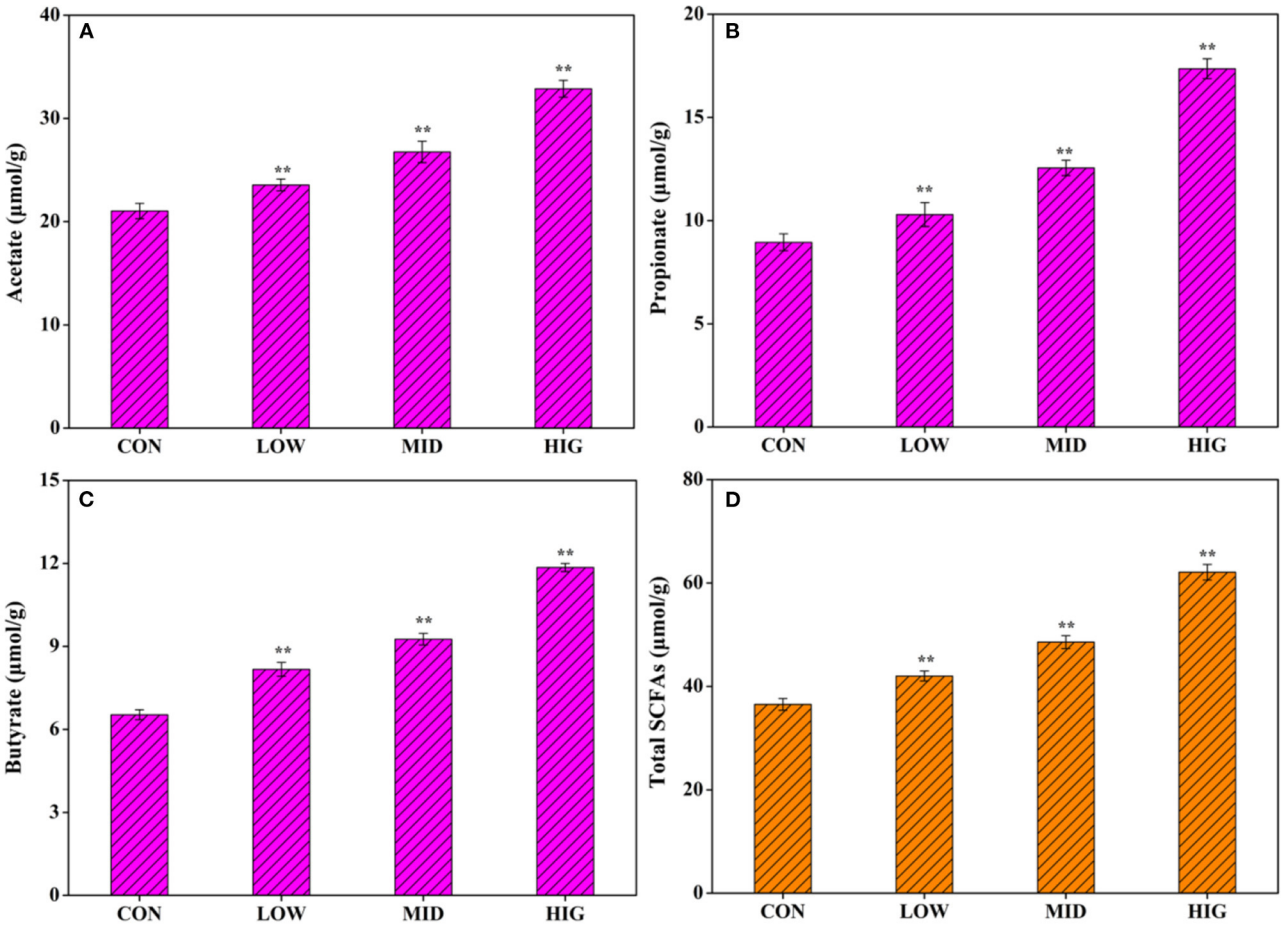
Influence of GPP on acetate, propionate, butyrate, and total SCFAs. **(A)** Acetate, **(B)** propionate, **(C)** butyrate, and **(D)** total SCFAs. CON: control group, LOW: 100 μg/ml GPP, MID: 400 μg/ml GPP, HIG: 800 μg/ml GPP. Data are expressed as mean ± SD (*n* = 6). Significance was determined through ANOVA, *P < 0.05, **P < 0.01.

Non-starch polysaccharides are resistant to saliva, succus gastricus, and succus entericus, which will be digested by the gut microbiota into SCFAs to exert biological activities (26). As shown in Figure 2A, after administration of different concentrations of GPP for 3 weeks, the acetate concentration in the cecum contents increased from 21.03 ± 0.75 μmol/g (CON) to 23.55 ± 0.58 μmol/g (LOW), 26.75 ± 1.03 μmol/g (MID), and 32.88 ± 0.82 μmol/g (HIG). Figure 2B shows that the propionate concentration increased from 8.95 ± 0.41 μmol/g (CON) to 10.29 ± 0.53 μmol/g (LOW), 12.55 ± 0.37 μmol/g (MID), and 17.36 ± 0.48 μmol/g (HIG). Figure 2C indicates that the butyrate concentration increased from 6.53 ± 18 μmol/g (CON) to 8.17 ± 0.25 μmol/g (LOW), 9.26 ± 0.21 μmol/g (MID), and 11.85 ± 0.15 μmol/g (HIG). Figure 2D illustrates that the level of total SCFAs increased from 36.51 ± 1.15 μmol/g (CON) to 42.01 ± 96 μmol/g (LOW), 48.56 ± 1.24 μmol/g (MID), and 62.09 ± 1.53 μmol/g (HIG). Furthermore, the increase in SCFAs was positively correlated with GPP concentration, with acetate having the highest increment.

Previously, Gao et al. (27) verified that when gellan gum was degraded to gellan oligosaccharide with molecular weight of 72,903 Da, the production of acetic acid and propionic acid by gellan oligosaccharide was increased in a bionic intestinal reactor *in vitro*, indicating that relatively low molecular weight is a key factor for polysaccharide to regulate gut microbiota for SCFAs production. Molecular weight of GPP used in present work was 40,700 Da (12), which might be a factor affecting its probiotic activity on increasing SCFAs production. SCFAs not only are important in energy supply and gut microbiota health but also might affect the metabolism or function of peripheral tissues *via* entering systemic circulation (28), such as body weight regulation. For instance, Canfora et al. (29) found that SCFAs could prevent and counteract obesity by promoting satiety. Other researchers reported that acetate in SCFAs plays an important role in body weight regulation (30). GPP reduced body weight possibly *via* increasing the level of SCFAs, especially acetate.

### Effect of GPP on Gut Microbiota

#### Influence of Gut Microbiota Diversity by GPP

Many statistical indices are used to assess the abundance and diversity of microbial communities. Ace and Chao1 indices are used to assess the number of operational taxonomic units (OTUs) in the gut microbiota; Simpson and Shannon indices reflect the species diversity of the gut microbiota (31). As shown in Table 2, the Ace and Chao1 indices of the gut microbiota in the experimental group increased compared with that in the control group in a concentration-dependent manner. Hence, the intake of GPP increased the species richness of the gut microbiota. The Shannon index of the gut microbiota showed an increasing trend, whereas the Simpson index had a downward trend. This finding suggested that the increase of species diversity of the gut microbiota was induced by administration of GPP (22). When Deng et al. (32) studied different molecular weights konjac glucomannans (KGM) on the hypoglycemic effects of type 2 diabetic rats, they found that medium molecular weights KGMs could increase gut microbiota diversity. Therefore, molecular weight (40,700 Da) might be one of the factors affecting gut microbiota diversity by GPP (12).

**Table 2:** Influence of GPP on the α-diversity of gut microbiota.

**Groups**	**Species richness**		**Species diversity**	
	**Chao1**	**Ace**	**Shannon**	**Simpson**
CON	1,970 ± 163^a^	2,018 ± 174^a^	6.58 ± 0.42^a^	0.98 ± 0.005^a^
LOW	2,116 ± 203^b^	2,209 ± 195^b^	7.39 ± 0.35^b^	0.97 ± 0.003^a^
MID	2,289 ± 314^c^	2,278 ± 269^b^	7.54 ± 0.51^b^	0.97 ± 0.003^a^
HIG	2,536 ± 295^d^	2,641 ± 357^c^	8.26 ± 0.48^c^	0.95 ± 0.002^a^

*CON, control group; LOW, 100 μg/ml GPP; MID, 400 μg/ml GPP; HIG, 800 μg/ml GPP. Data are expressed as mean ± SD (n = 6). Different letters represent significant differences, P < 0.05*.

**Figure 3:**
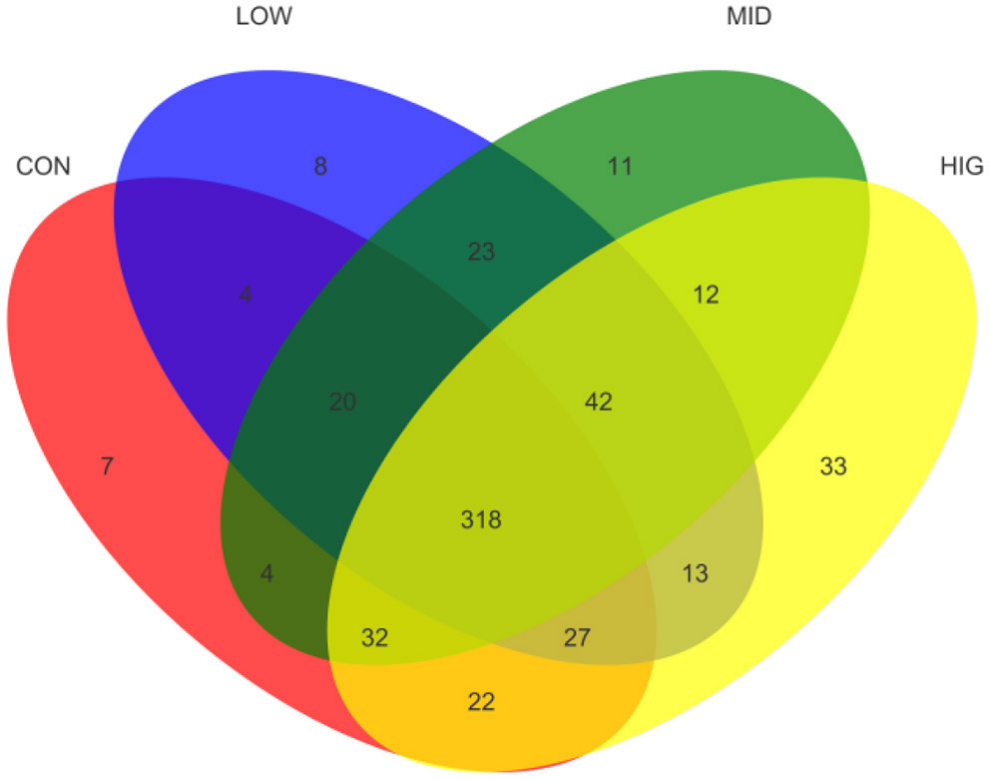
Venn diagram of the gut microbiota among different groups.

The effect of GPP on the species richness of the gut microbiota was detected by number of OTUs, and the results are shown as Venn diagram in Figure 3. The total numbers of OTUs in CON, LOW, MID, and HIG were 434, 455, 462, and 499, respectively. Among all OTUs, 318 were shared by four groups and 42 were only shared by three experimental groups. Meanwhile, the numbers of OTUs separately shared between control group and experimental groups were 369 (CON and LOW), 374 (CON and MID), and 399 (CON and HIG). In addition, each group had its own unique OTUs: 7 for CON, 8 for LOW, 11 for MID, and 33 for HIG. Hence, GPP improved the species richness and diversity of the gut microbiota.

#### Gut Microbiota Analysis at Phylum Level

**Figure 4:**
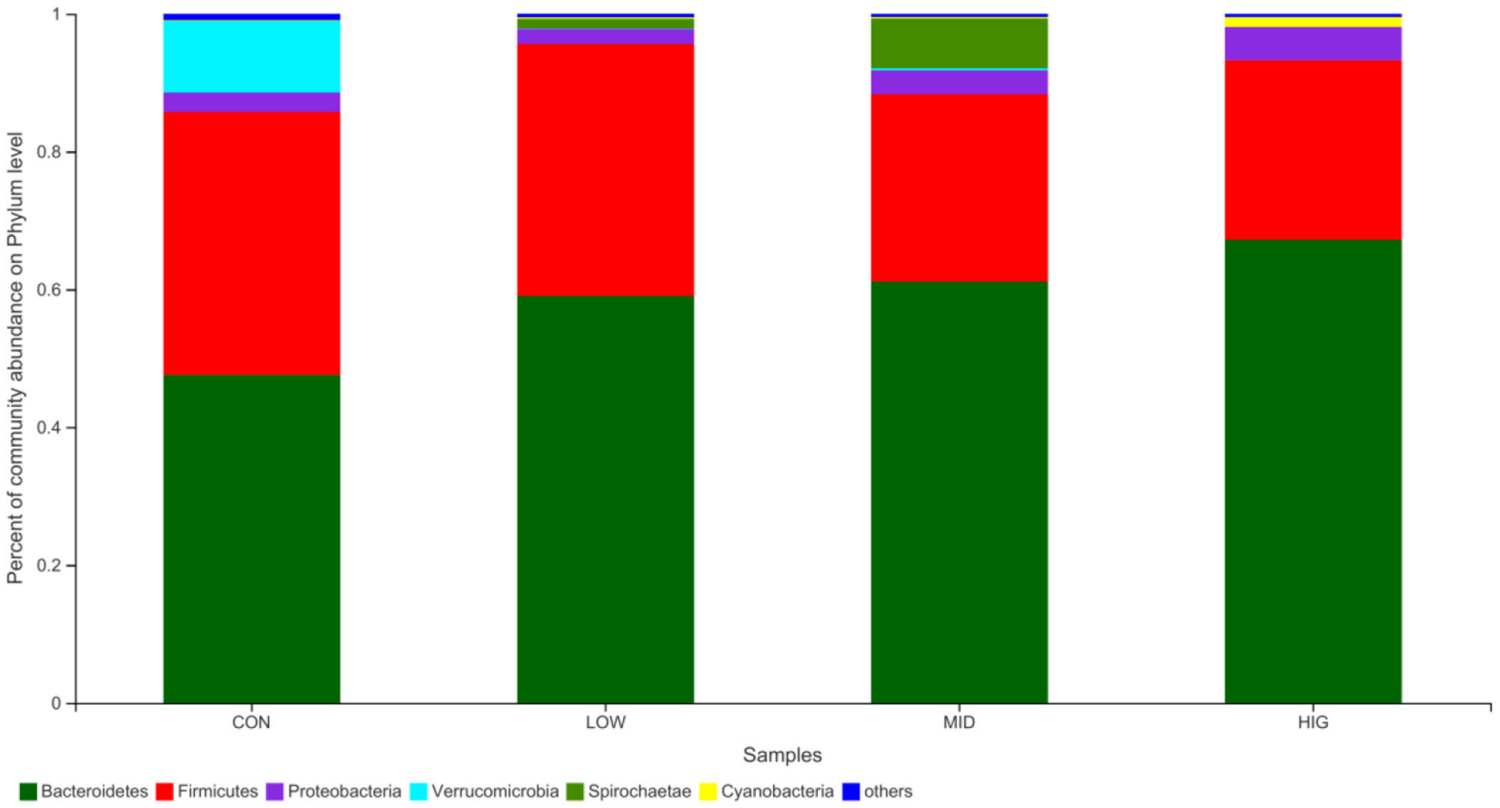
Relative abundance of the gut microbiota at phylum level.

The regulatory effect of GPP on the gut microbiota is shown in Figure 4. Among the four groups, the main bacteria (relative abundance > 1%) at the phylum level are Bacteroidetes, Firmicutes, Spirochaete, Proteobacteria, Verrucomicrobia, and Cyanobacteria, accounting for more than 95% of the sequences. Bacteroidetes and Firmicutes are the main bacteria that utilize undigested non-starch polysaccharides (33) and are involved in maintaining the balance of energy metabolism (25). In experimental mice, the relative abundance levels of Bacteroidetes were 47.58% (CON), 59.04% (LOW), 61.13% (MID) and 67.21% (HIG), and those of Firmicutes were 38.16, 36.58, 27.20, and 26.03%, respectively. The F/B ratios in the four groups were 0.8021, 0.6196, 0.4450, and 0.3873, indicating a decreasing trend. Bacteroidetes possess genes for encoding succinate pathway and are the primary polysaccharide-degrading and propionate-producing bacteria in gut (34), this might be the reason of SCFAs increase in gut induced by F/B ratio decrease and Bacteroidetes increase.

Many structural characteristics could affect the probiotic activity of polysaccharides. For instance, Yu et al. (34) reported that the monosaccharide order of sugar beet pulp polysaccharide used by gut microbiota might arabinose, glucose, fucose and galacturonic acid. Zhao et al. (35) found that glycosidic bond type could not only affect the body weight of mice, but also the composition and proportion of mice gut microbiota. Deng et al. (32) suggested that molecular weight of konjac glucomannan might affect its effect on gut microbiota diversity. Gong et al. (36) demonstrated that uronic acid could affect the biological activity of polysaccharides. GPP was composed of rhamnose, arabinose, galactose, glucose, xylose, mannose, galacturonic acid and glucuronic acid in a molar ratio of 4.11: 7.34: 13.31: 20.99: 1.07: 0.91: 4.75: 0.36, and its weight-average molecular weight was 4.070 × 104 Da (12). Arabinose, glucose and uronic acid in its monosaccharide composition and moderate molecular weight (4.070 × 104 Da) might be the reason for probiotic activity of GPP.

Although Gao et al. (37) found that there was no direct relationship between F/B ratio and body weight regulation, many researchers suggested that an increase in the F/B ratio induced obesity and prevented body weight loss. Zhao et al. (22) reported that the body weight reduction effect of *Auricularia auricular* polysaccharide might be related to decrease in the F/B ratio. Ley et al. (33) demonstrated that an increase in Firmicutes and a decrease in Bacteroidetes are likely to induce obesity. Chang et al. (38) and Koliada et al. (39) suggested that the reduction in the F/B ratio could lead to weight loss and improved body health. Therefore, the decreased F/B ratio may lead to decrease in body weight gain caused by GPP.

## Conclusion

In present work, a polysaccharide fraction of GPP extracted from *Gynostemma pentaphyllum* was used to detect its effect on the body weight and gut microbiota of normal mice. With administration of GPP once a day for 3 weeks, body weight gain of normal mice reduced but SCFAs levels in gut increased. Meanwhile, GPP increased gut microbiota diversity but decreased Firmicutes/Bacteroidetes ratio in the gut of normal mice. However, the mechanism through which the polysaccharide affects the gut microbiota and the type of bacteria that participate in the degradation of polysaccharides remain unclear.

## Data Availability Statement

The original contributions presented in the study are included in the article/supplementary material, further inquiries can be directed to the corresponding author/s.

## Ethics Statement

The animal study was reviewed and approved by the Animal Ethics Committee of Henan University of Technology.

## Author Contributions

SL contributed to conception, design, and funding of the study. YW, WD, and WH organized the database. TN wrote the first draft of the manuscript. QS and ZW contributed to writing—review and editing. All authors contributed to the article and approved the submitted version.

## Funding

This work was supported by the Science and Technology Research Project of Henan Province (182102310687), Henan Provincial Education Department Project (19B550010), the Natural Science Foundation of Henan Province (212300410131), and the Natural Science Foundation of Chongqing (cstc2019jcyj-msxmX0459).

## Conflict of Interest

The authors declare that the research was conducted in the absence of any commercial or financial relationships that could be construed as a potential conflict of interest.

## Publisher's Note

All claims expressed in this article are solely those of the authors and do not necessarily represent those of their affiliated organizations, or those of the publisher, the editors and the reviewers. Any product that may be evaluated in this article, or claim that may be made by its manufacturer, is not guaranteed or endorsed by the publisher.
